# Integrating omics data and protein interaction networks to prioritize driver genes in cancer

**DOI:** 10.18632/oncotarget.19481

**Published:** 2017-07-22

**Authors:** Tiejun Zhang, Di Zhang

**Affiliations:** ^1^ GMU-GIBH Joint School of Life Sciences, Guangzhou Medical University, Guangzhou, Guangdong 511436, China; ^2^ School of Computer Science and Technology, Anhui University, Hefei, Anhui 230601, China

**Keywords:** driver genes, protein interaction network, integrative data

## Abstract

Although numerous approaches have been proposed to discern driver from passenger, identification of driver genes remains a critical challenge in the cancer genomics field. Driver genes with low mutated frequency tend to be filtered in cancer research. In addition, the accumulation of different omics data necessitates the development of algorithmic frameworks for nominating putative driver genes. In this study, we presented a novel framework to identify driver genes through integrating multi-omics data such as somatic mutation, gene expression, and copy number alterations. We developed a computational approach to detect potential driver genes by virtue of their effect on their neighbors in network. Application to three datasets (head and neck squamous cell carcinoma (HNSC), thyroid carcinoma (THCA) and kidney renal clear cell carcinoma (KIRC)) from The Cancer Genome Atlas (TCGA), by comparing the Precision, Recall and F1 score, our method outperformed DriverNet and MUFFINN in all three datasets. In addition, our method was less affected by protein length compared with DriverNet. Lastly, our method not only identified the known cancer genes but also detected the potential rare drivers (*PTPN6* in THCA, *SRC*, *GRB2* and *PTPN6* in KIRC, *MAPK1* and *SMAD2* in HNSC).

## INTRODUCTION

With the development of high throughput sequencing, amounts of cancer omics data have allowed us to better understand cancer biology [1]. The Cancer Genome Atlas (TCGA) project stores omics data of more than 20 cancer types, thus allows us to study cancer driver genes (driver gene is a specific type of cancer gene). However, the key question is how to distinguish the driver genes, which confer a selective advantage to tumor growth, from passengers, which provide no fitness advantage to the tumor [2]. Besides, how to subsequently integrate omics data, including exploit protein interaction networks to detect cancer driver genes remains a challenge. Computational approaches and tools have been developed to identify driver genes. These methods can be categorized into gene level and module level approaches [3]. Gene level approaches for identifying drivers mainly rely on the hypothesis that driver has a more chance to be mutated across a set of tumors [4, 5]. These approaches including the mutational significant in cancer (MuSiC) [6], OncodriverCLUST [7], and MuSigCV [8], which can identify the genes that harbor significantly more mutations than background mutation rate. Although the gene level approaches can be used to distinguish driver genes from passengers, rare mutations played functional roles in later stages of tumor progression are failed to be detected [9]. What's more, cells are made up of multiple molecular structures that form dynamic networks [10]. Under a network, a genetic aberration may affect its connection within the network [10]. The module level approaches using the network or pathway information can be effective in identifying drivers. An example is that Hamed and his colleges used the protein interaction network for identifying cancer drivers [11]. DawnRank, which used PageRank algorithm to detect driver genes [12], is an affective module level method. Besides, DriverNet identifies drivers by estimating their effect on mRNA expression [13]. MUFFINN prioritizes driver genes based on genes and their neighbors in functional network [14]. In addition, some approaches were developed to identify driver modules or pathways, such as MEMCover and MEMo [15, 16]. Although these approaches are effective in detecting drivers, they have limitations. First, the network is built based on static network, rather being condition specific. Second, network edges are often not considered in the majority of the aforementioned approaches.

In view of the functional relationship between gene pairs in a network may radically boost the detection of drivers [17]. The network consists of nodes (which may stand for genes or proteins) and edges (which may present the functional links that connect them). Merid et al. performed a network-based algorithm to identify driver genes by considering the relationship between the mutation events and functional gene sets (FGS), the result showed a complementary to frequency-based driver analyses [17]. However, their method did not consider gene expression data. In a biological network, an interacting gene pair tends to present positive or negative correlations which are reflected by the gene expression data. Alteration of these gene correlations indicates system's condition (such as normal or disease state) [18]. Therefore, differentially correlated gene pairs can distinguish tumor and normal sample [19]. Thus, candidate potential cancer genes can be detected by studying dynamic regulation between genes, which may improve the detection of driver genes [17].

In this paper, we presented a network-based approach which integrated gene expression, mutation data, and PPI (protein-protein interaction) data to distinguish between driver and passenger genes. Our approach built on a hypothesis that a driver gene can be determined by its neighbors. We firstly built a relationship network among the DCGs (differentially coexpressed genes), functional genes, and then calculated the impact of DCGs by weighting the relationship between the DCGs and its connective functional genes on a bipartite graph. Finally, we combined the mutation information to improve the effect of screening drivers. In order to evaluate the performance of our approach, we applied it to three datasets (KIRC (kidney renal clear cell carcinoma), THCA (thyroid carcinoma), and HNSC (head and neck squamous cell carcinoma)) to identify driver genes. We detected some potential rare drivers that previously could not be identified by DriverNet such as *SMAD2* in HNSC [13]. Besides, we also detected some known cancer driver genes in each cancer dataset. All in all, our computational method is effective to detect potential cancer drivers to improve cancer-specific therapeutic targets.

## RESULTS

### Performance comparison

**Figure 1:**
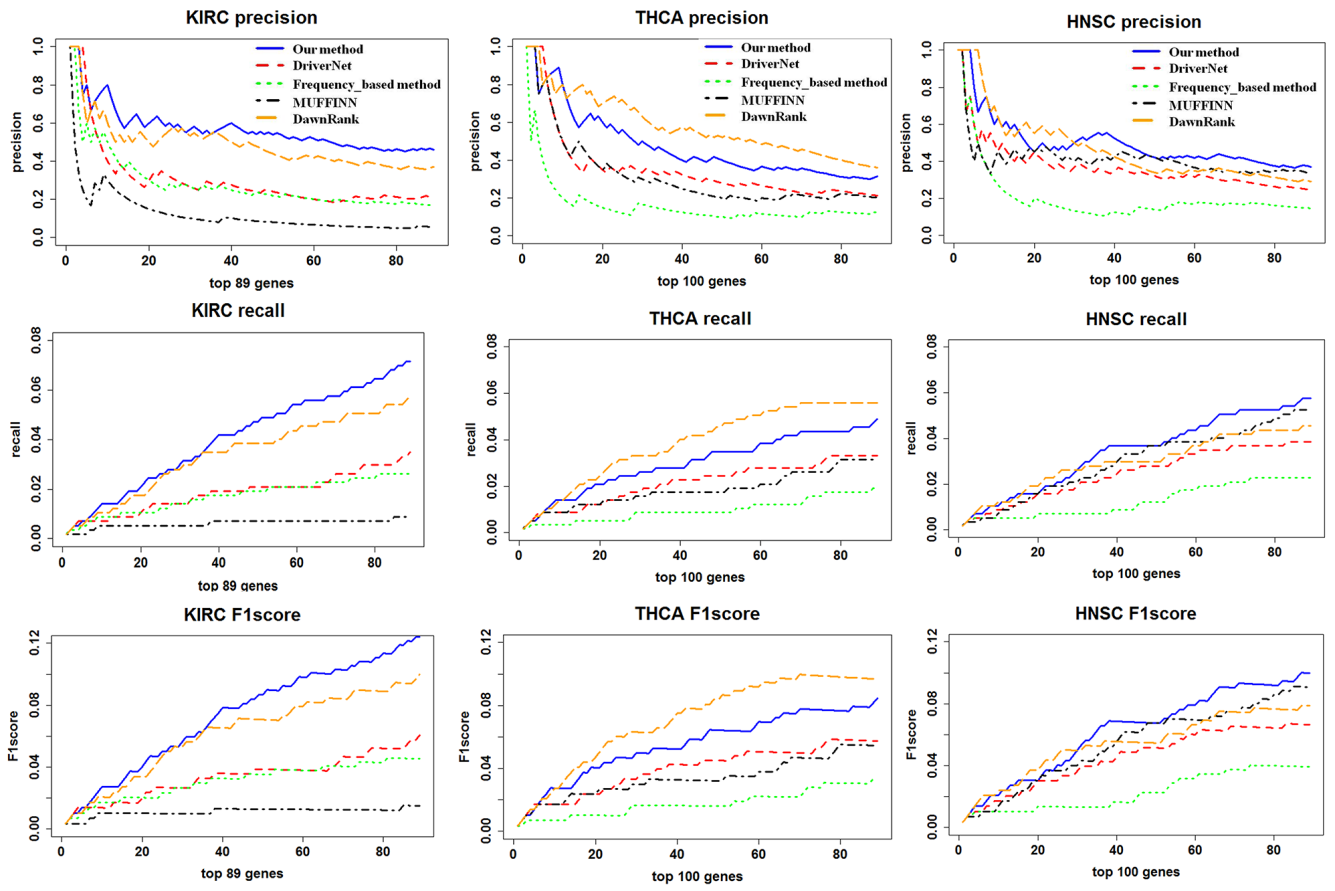
A comparison of the precision, recall, and F1 score for the top ranking genes in our method and DriverNet The X-axis represents the number of top ranking genes involved in the precision, recall, and F1 score calculation. The Y-axis represents the score of the given metric.

In order to assess the performance of our method's ability to detect known driver genes, DriverNet and DawnRank and MUFFINN, and frequency-based methods were used to be compared with our method in CGC (the Cancer Gene Cense database) and driver gene list defined by 20/20 rules [20] as benchmark of known drivers. We performed the comparisons as follows: we used the same datasets to perform the DriverNet, DawnRank, MUFFINN, and frequency-based method and our method, respectively. We input these datasets into DriverNet, DawnRank, and MUFFINN. Then ran the program with the default settings, and we ran our method with the settings mentioned above. We calculated the DCGs Z-score and the mutated gene Z-score respectively. Combining the two scores and using the total score as the driver gene score. In CGC benchmark dataset, to evaluate the comparison, we used the three measures (Precision, Recall, and F1 score) mentioned in the Method Section. Based on these measures, our approach showed a better performance than MUFFINN and DriverNet and frequency-based method. We first evaluated the performance of our method. In Figure 1, Precision, Recall and F1 score curves of our method are both higher than those with DriverNet, MUFFIN and frequency-based method, but slightly worse than those with DawnRank method in THCA and HNSC datasets. Although DriverNet performed comparably in ranking the top 5 genes in THCA and KIRC, it has poorer performance in all driver genes. A potential explanation of the difference may lie in the CGC is not cancer-specific and the cancer gene listed in CGC is not complete. In Table 1, we can observe *LYN* is not a CGC gene, while they have been reported to have an association with THCA [21].

**Table 1:** Top 5 cancer–associated driver genes in each three cancer type

	Ranking	Gene	Driver gene score	Annotation	The number of mutated gene
THCA	1	EGFR	11.58	Sanger cancer gene	3
	2	EP300	11.20	Sanger cancer gene	3
	3	NRAS	9.97	Sanger cancer gene	31
	4	LYN	9.78		3
	5	PTPN11	9.42	Sanger cancer gene	11
HNSC	1	TP53	39.63906	Sanger cancer gene	172
	2	PIK3CA	19.19902	Sanger cancer gene	100
	3	EGFR	16.93629	Sanger cancer gene	40
	4	EP300	16.62027	Sanger cancer gene	22
	5	FADD	15.40049		81
KIRC	1	PBRM1	41.84646	Sanger cancer gene	138
	2	SETD2	14.98057	Sanger cancer gene	51
	3	BAP1	11.83806	Sanger cancer gene	42
	4	SRC	11.66549		2
	5	EP300	11.39506	Sanger cancer gene	6

**Figure 2:**
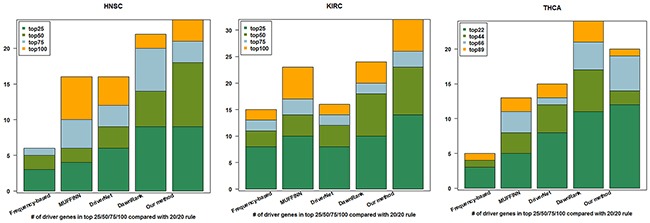
Cumulative numbers of retrieved cancer genes annotated by 20/20 rule within top 25, 50, 75, and 100 of HNSC and KIRC, top 22, 44, 66, and 89 of THCA using four different methods

In benchmarking 20/20 rule dataset, we used the top ranked driver genes (top 89, 100, and 100 driver genes for THCA, HNSC, and KIRC, respectively (see Supplementary Table 1) to compare with other methods, which is shown in Figure 2. It can be seen that our method outperformed the other four methods on the top 100 genes in HNSC and KIRC dataset. In THCA, our method has a remarkably better performance than frequency-based method, MUFFIN, and DriverNet, but slightly worse than DawnRank. However, in the top 22 gene list, our method presents advantage than DawnRank.

For THCA, our method identified 89 genes (driver gene score ≥2) which includes 28 genes found in CGC. 11 genes found in CGC (*NRAS*, *HRAS*, *PTPN*, *PTEN*, *RB1*, *EP300*, *ATM*, *PIK3R1*, *TP53*, *HSP90AA1*, *PML*) were also among the driver genes nominated by DriverNet approach. *TG* (thyroglobulin) is the 13th-ranked driver in our method, and it is altered in 20/435 THCA samples. *TG* plays a role in the pathogenesis of papillary thyroid carcinoma and its malignant evolution [22]. However, it ranked 478 and was not detected by DriverNet as top ranking drivers.

For KIRC, DriverNet identified 100 driver genes, of which 21 driver genes were found in CGC. Our method identified 127genes (driver gene score ≥2), 52 genes out of them were found in CGC. 12 genes found in CGC (*PTEN*, *TP53*, *EGFR*, *EP300*, *SMARCA4*, *ATM*, *CREBBP*, *APC*, *NCOR1*, *XPO1*, *AKT1*, *HSP90AA1*) were also among the driver genes nominated by DriverNet. We detected *PBRM1* as the first ranked gene (mutated in 138 cases) [23], whereas it was not detected by DriverNet.

For HNSC, DriverNet identified 202 driver genes, of which 23 were found in CGC. Our method identified 202 genes (driver gene score≥2), among them, 49 genes were included in CGC. 17 genes (*TP53*, *PIK3CA*, *EGFR*, *EP300*, *CREBBP*, *NOTCH1*, *SMAD4*, *FGFR1*, *HRAS*, *CASP8*, *NFE2L2*, *MDM2*, *RAC1*, *HSP90AA1*,*AKT1*, *PLCG1*, and *CDH1*) found in CGC were also among the driver genes nominated by DriverNet. *TP53*, *PIK3CA*, *HRAS*, and *EGFR* are known oncogenic drivers and it indicates that the efficiency of integrating multi-omics data to detect drivers. In addition, *STAT3* mediates the cell cycle, regulates apoptosis, which has been reported to be constitutively active in HNSC [24]. However, *STAT3* which was ranked 27th was not found by DriverNet.

Our method is less affected by noise than DriverNet. An illustration is that *TTN* was ranked 23th in KIRC and 18th in HNSC by DriverNet. *TTN* is the longest gene in the human genome, which has a higher mutation rate and a potential to be artifacts [25]. However, *TTN* was not detected as driver genes in any three cancers according to our method.

### Infrequent (rare) driver mutations identified in three cancer types (THCA, KIRC, and HNSC)

In this section, we validated the ability of detecting rare drivers by our method. We adopted the three criteria to identify rare driver genes. Firstly, only the top 30 of driver genes in various samples were considered as drivers. Secondly, the alteration frequency should be lower than 2% in tumor samples. And finally the gene should not be reported in CGC [12].

In THCA, we found some novel rare driver genes. Of them, *PTPN6* is the most promising. *PTPN6* has been described as a tumor suppressor gene [26]. *PTPN6* participates in several cancer related pathways, including adherens junction, T cell receptor signaling pathway, B cell receptor signaling pathway, and Jak-STAT signaling pathway (see Supplementary Table 2). *PTPN6* is the 11th-ranked driver in our method, and it is altered in 0.23% THCA samples.

**Figure 3:**
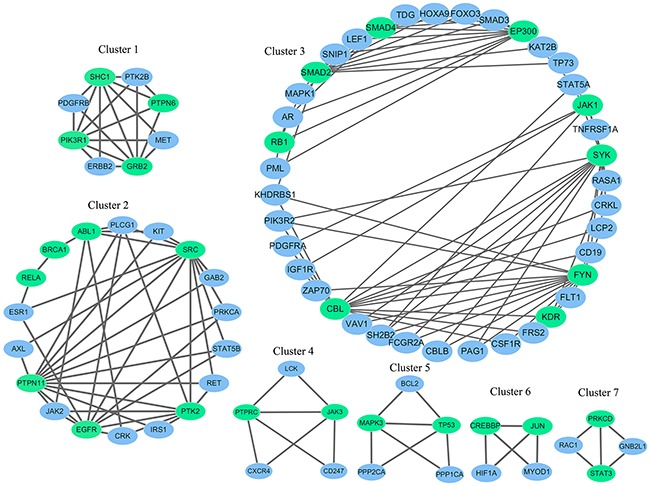
The gene modules identified using the top 50 genes and their corresponding interaction partners in KIRC Genes in green ellipse represent the detected driver gene, while genes in blue ellipse represent the driver genes’ interaction partners.

In KIRC, there were three candidate novel drivers including *SRC*, *GRB2* and *PTPN6*. *SRC* is 5th-ranked driver in our method, and it is altered in 0.48% KIRC samples. *SRC* is human proto-oncogene, which was reported as a novel therapeutic target in renal cell carcinoma [27]. *GRb2* (The adapter protein growth factor receptor-bound 2), a scaffolding adaptor protein, has recently been involved in a critical crosstalk between RTK signals and the intracellular signals [28]. The enrichment analysis shows that *GRb2* participates in multiple cancer related pathway, such as chemokine signaling pathway, ErbB signaling pathway, MAPK signaling pathway, Jak-STAT signaling pathway (see Supplementary Table 2). There is a significant association between the protein tyrosine phosphatase PTPN6 (SHP-1) and GRB2 expression, which may amplify tyrosine kinase signaling in human breast cancer [29]. Nevertheless, direct demonstration of the relationship between the PTPN6 (SHP-1) and GRB2 in KIRC has been reported. In the cluster 1 of the Figure 3, we can observe that *PTPN6* and *GRB2* connect with each other. *GRB2* is the 6th-ranked driver in our method, and it is altered in 0.24% KIRC samples. *PTPN6* is the 12th-ranked driver in our method, and it is altered in 0.72% HNSC samples. These findings suggest potential crosstalk between mutant *PTPN6* and *GRB2*.

In HNSC, two potential novel drivers we found are *MAPK1* and *SMAD2*. *SMAD2* is 28th-ranked driver in our method, and it is altered in 0.59% HNSC samples. The algorithm also identified a well-established tumor suppressor gene *SMAD2*. According to the pathway enrichment analysis, *SMAD2* is involved in TGF-beta signaling pathway, Cell cycle, and Wnt signaling pathway (see Supplementary Table 2). In addition, *SMAD2* mutations in human head and neck cancer have been reported [30]. Additionally, we identified one MAP kinase *MAPK1*, which is 19th-ranked driver in our method, and it is altered in 0.79% HNSC samples. We can observe that *MAPK1* is involved in multiple pathways (see Supplementary Table 2). In a recent study, MAPK1 (p38) mediates epithelial-mesenchymal transition to drive HNSC metastasis [31].

### Confirmation of cancer genes

**Figure 4:**
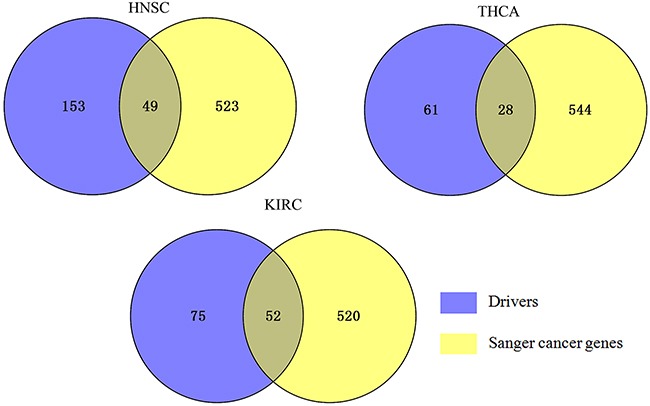
Overlap of the known cancer genes identified for three cancers Venn diagram represents the overlap between each cancer-specific driver genes and CGC. 572 known cancer genes were obtained from the CGC database, 49, 28, 52 of which appear in HNSC drivers, THCA drivers, and KIRC drivers respectively.

In total, we found 89, 127, and 202 drivers in THCA, KIRC, and HNSC respectively (see Supplementary Table 1). The identified driver genes were overlapped with CGC and shown in Figure 4. In THCA, it can be seen that 28 driver genes out of top 89 driver genes are known driver genes in CGC (p-value < 2.2e-16). In HNSC, of these top 202 driver genes, 49 driver genes are in CGC (p-value < 2.2e-16). In KIRC, of these top 127 driver genes, 52 are identified in CGC. Our result indicates that the detected driver genes are enriched among known cancer related genes and cannot be selected randomly.

We examined the top 5 ranked in THCA, HNSC, KIRC respectively (Table 1). In addition, 12 genes have been functionally linked to cancer in multiple reports.

In THCA, *EGFR* is especially intriguing. It is a member of the protein kinase superfamily, and ranked first in the predicted THCA driver genes. *EGFR* mediated downstream signal transduction and was overexpressed in an aplastic thyroid cancer cell lines, rendering this receptor a potential target for molecular therapy [32]. The third-ranked gene, *NRAS*, encodes membrane-associated proteins that play a vital role in the transduction of signals [33], which has been reported in thyroid cancer [34]. The *EP300* gene encodes p300, which is significant in the processes of cell proliferation and differentiation [35]. It has altered protein expression in thyroid cancer [36]. *LYN* has been previously reported in thyroid cancer [21]. *PTPN11* encodes protein-tyrosine phosphatase SHP2, and their domains are involved in cellular signaling. Besides, PTPN11 was significantly increase expressed in human thyroid carcinoma [37]. All told, all the top 5 predicted driver genes have evidenced in the literature for their roles in THCA.

In HNSC, *TP53* was the top ranked driver in our analysis. Three (*TP53*, *PIK3CA*, and *EGFR*) of the top 5 genes were previously known HNSC genes [38]. Daniel Martin and his colleges suggested that *EP300* genomic alternations may promote HNSC initiation and progression [39]. It is noted that *FADD* was ranked 5th in our result. *FADD* can recruit other proteins to active NFκB and MAPK pathways [40]. It was overexpressed and considered to be a driver gene in HNSC [41].

In KIRC, *PBRM1* was the top ranked driver in our analysis. *PBRM1*, *SETD2*, and *BAP1* were previously known HNSC genes and recently found to be altered in clear cell renal cell carcinoma [23, 42]. They are highly mutated (Table 1). *SRC* was identified as a novel rare gene. It is one of the markers for low-grade renal cell carcinoma [43]. *EP300* is ranked 5th (Table 1) and we observed that it is mutated in 6 KIRC samples. It has been reported that *EP300* behaves as a classical tumor-suppressor gene in human cancers [35].

*EP300* was identified as a top gene in three datasets simultaneously (Table 1). It was 25th-ranked, 14th-ranked, and 9th-ranked in THCA, HNSC, and KIRC respectively by DawnRank method. In DriverNet, it seems the same situation. *EP300* was 16th-ranked, 15th-ranked, 19th-ranked in THCA, HNSC, and KIRC respectively by DriverNet method. These results further suggest that the important roles of *EP300* in cancers.

## DISCUSSION

In recent years, various computational approaches and tools have been developed to identify drivers, however, there are some limitations in detecting driver genes, for example, DriverNet has a bias toward long mutated genes. In two benchmark datasets, our method outperforms DriverNet, MUFFINN and frequency-based method in all three cancer types, although the performance of DawnRank is slightly higher than that of our approach in one of three databases. Our method is simple and parameter free. Meanwhile, the result indicates that our pipeline shows its ability to detect driver genes, even rare driver genes. Two potential reasons may contribute to the result. First, we consider the dynamic of network. Second, we construct a bipartite graph, which represents the relationship between the DCGs and the functional genes impacted by them in network. If a DCG impacts the more functional genes, the more likely it may be the driver. Our method has the following advantages. Firstly, this approach detects common and known drivers with a better performance than previous method. Secondly, it can filter out long genes with a higher mutation rate, such as the TTN gene which can't contribute to cancer. Thirdly, it can find some potential co-expression gene pairs, for instance, *PTPN6* and *GRB2* in KIRC. Our benchmarking analysis suggests that our algorithm is robust to noise and works well in three TCGA cancer types, making it general to different cancer types if the mutation data and gene expression data are available. In essence, the approach demands information on the context of the DCG (differentially coexpressed gene) of interest, functional gene set that constitute known cancer related pathways, and the connections between genes in the global network. There are also, however, limitations. One of the limitations is that HPRD is not large enough, DCGs or functional genes not in the PPI were filtered out and it may ignore some candidates. This can be improved with the growth of the database of HPRD in the future. Another limitation is that the network used in our method is not patient-specific or cancer-specific. Therefore, perturbations specific to patient may be obscured by this pipeline. The last limitation is that our approach detects potential drivers rely on the common effect of DCGs altering the functional gene. However, this may not be the case for all drivers. As more and more efforts are being devoted into understanding cancer genomes, we expect that our method's ability to detect drivers would also improve.

Taken together, we developed a practical analysis pipeline to predict potential driver genes in cancer. Although this study focuses on THCA, KIRC and HNSC, the method is broadly applicable to any other cancer types for which mutation and expression data are available. In addition, our results demonstrate the efficiency of integrative analysis across three cancer types, not only the known cancer genes were identified, but also the potential rare drivers were detected. In future, we will combine the gene expression, copy number variation, and methylation data to construct a heterogeneous network. In addition, we will apply machine learning method to improve the performance. We expect this approach can generalize well to perform the future studies, including determine the optimal treatment tactics for each patient through integrating patient-specific omics data.

## MATERIALS AND METHODS

### Datasets and pre-processing

The RNASeqV2 data (level three), gene mutation data (level two) and CNV data were downloaded from TCGA data portal. RNAseq expression levels, available as RSEM (RNAseq by Expectation Maximization) were transformed to log2 (RSEM+1). The GISTIC (version 2) was applied to the DNA copy number data. The information of three cancer types used in our method was provided in Table 2. For gene expression dataset, NA values were replaced by mean value.

**Table 2:** Overview of the number of samples for three cancer types with gene expression and mutation data

Cancer type	Tumor expression samples	Normal expression samples	Somatic mutation samples
KIRC	534	72	417
HNSC	522	44	509
THCA	513	59	435

### Mutation matrix

Mutation matrix combined somatic mutation data and CNV data by extracting genes from deleted and amplified fragments in CNV data. The common samples between the mutation data and CNV data were retained. Mutation matrix (i, j) is a binary matrix where M (i, j) =1 indicates sample j have a gene i mutated and M (i, j) =0 indicates sample j don't have a gene i mutated.

### Differentially coexpressed genes

Differential co-expression analysis is designed to examine the alternation in gene expression correlation between the tumor samples and the normal samples, which is developed as a complementary approach to traditional differential expression analysis [44]. DCGs were obtained by using Differential coexpression profile (DCp)function in DCGL (differentially coexpressed genes and links) package [44]. We used the Pearson Correlation Coefficient (PCC) to measure the relationships between the expression profiles of all gene pairs and calculated the false discovery rate (FDR) by Benjamini–Hochberg method to adjust the raw p values [45]. Gene with threshold of FDR less than 0.25 were selected as DCGs [46].

### Network construction and functional gene sets

The network is an undirected graph G (V, E) where V stands for the genes and edges (i, j) E are weighted by PCC. Protein interaction network was sourced from HPRD (http://www.hprd.org) database and protein self-interactions were removed, resulting in 39240 interactions among 9616 proteins. Functional gene sets (FGS) were obtained from [47], which contain all of the KEGG pathways [48] and 15 GO terms [49], which could be related to hallmarks of tumor [50]. Then a matrix was used to leverage to DCGs to their consequent effect on functional gene. The associations between DCGs and functional genes were built using a bipartite graph where left nodes represent DCGs and right nodes stand for functional genes. We formulate the network with DCGs = {g_1_,g_2_,…g_n_}, functional genes = {g_1_,g_2_,…g_m_}. Nodes g_n_ in the left partition and nodes g_m_ in the right interact have an edge, if g_g_ is DCG, g_m_ is a member of functional gene set, and g_n_ and g_m_ interact according to known PPI network.

### Details of our algorithm

**Figure 5:**
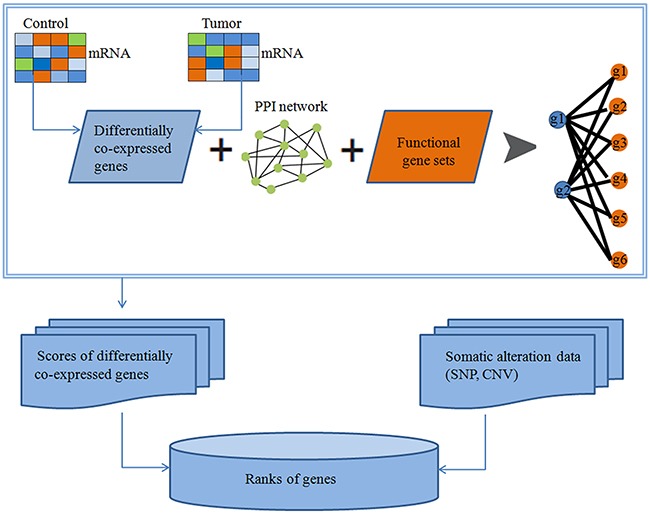
Identification of cancer related genes based on protein-protein interactions (PPIs)

In this study, we developed a pipeline to identify drivers. Figure 5 shows the schematic overview of approaches used in our study. Firstly, using the DCp function in DCGL package, we picked out DCGs. To improve statistical confidence, DCGs must have FDR<0.25. Secondly, we computed the DCGs score (Z-score) as:

$$\text{z}=\frac{d_{\text{AF}}-\mu_{\text{AF}}}{\sigma_{\text{AF}}},$$

where d_AF_ is the total score of weighted network between genes in the DCGs and the FGS, μ_AF_ is the expected mean of d_AF_, and σ_AF_ is the standard deviation of d_AF_. Thirdly, in order to improve the accuracy of detecting driver genes, mutation information was combined. Therefore, we calculated the number of each mutated gene in mutation matrix. We normalized it and got the mutated gene Z-score. Finally, the driver gene score was assigned by summing the corresponding DCG Z-score and mutated gene Z-score. We only considered genes with driver gene score ≥2 as potential driver genes.

### Performance benchmarking analysis of our method

In order to metric the efficiency of our results, we took CGC (http://cancer.sanger.ac.uk/cancergenome/projects/census/) as a benchmark to evaluate the effect of our method. In practice, the gold standard of known drivers is impractical in the absence of ground truth. However, well-studied CGC provides an approximate benchmark of known drivers [12, 13]. Besides, we consider additional dataset to assess the method. As defined by the 20/20 rule, only 138 driver genes have been discovered to date. Both of these datasets were used to assess the accuracy of our method. We compared our method with the DriverNet, DawnRank, frequency-based method and MUFFINN method.

In accordance with both approaches mentioned above, we used the same datasets to perform the analysis and restricted the comparisons to three tumor types only (HNSC, KIRC, and THCA). We adopted three measures (Precision, Recall and F1 score) as follows:

$$\text{Precision}=\frac{\left(\text{\#Mutated genes in CGC}\right)\cap \left(\text{\#Genes found in our method}\right)}{\left(\#\text{Genes found in our method}\right)}$$

$$\text{Recall}=\frac{\left(\#\text{Mutated genes in CGC}\right)\cap \left(\#\text{Genes found in our method}\right)}{\left(\#\text{Mutated genes in CGC}\right)},$$

$$\text{F1 score}=2\times \frac{\text{Precision}\times \text{Recall}}{\text{Precision}+\text{Recall}}.$$

### Significance estimation of the potential driver genes

In order to evaluate the significance of the identified driver genes, we performed a hypergeometric test to calculate the probability of a random overlap:

$$\text{P}\left(\text{X}\geq \text{x}\right)=1-\overset{\text{x}-1}{\underset{\text{k}=0}{\sum }}\frac{\left(\begin{array}{c} \text{M}\\ \text{k} \end{array}\right)\left(\begin{array}{c} \text{N}-\text{M}\\ \text{n}-\text{k} \end{array}\right)}{\left(\begin{array}{c} \text{N}\\ \text{n} \end{array}\right)},$$

where N is the total number of genes, M and n are the number of genes in two sets, and k is the number of the overlapped genes of the two sets.

### Functional enrichment analysis of driver genes and recognition modules

In order to annotate driver genes detected in our result, we used the online DAVID [51] website and observed significant enrichment of these genes in the term of KEGG pathway. Briefly, KEGG pathway terms were annotated to statistical significance in the gene set. Enrichment was calculated through the hyper-geometric test using a FDR less than 0.05. Molecular Complex Detection (MCODE) [52] that detects densely connected regions in large protein interaction networks were used to recognize modules. MCODE weights all nodes depended on their local network density by setting the highest k-core of the vertex neighborhood. We set the highest k-core is 2.

## SUPPLEMENTARY MATERIALS FIGURES AND TABLES







## References

[1] Meyerson M, Gabriel S, Getz G (2010). Advances in understanding cancer genomes through second-generation sequencing. Nat Rev Genet.

[2] Greenman C, Stephens P, Smith R, Dalgliesh GL, Hunter C, Bignell G, Davies H, Teague J, Butler A, Stevens C (2007). Patterns of somatic mutation in human cancer genomes. Nature.

[3] Yang L, Feng T, Hu Z, Delisi C (2015). Evaluation and integration of cancer gene classifiers: identification and ranking of plausible drivers. Sci Rep.

[4] Sjöblom T, Jones S, Wood LD, Parsons DW, Lin J, Barber TD, Mandelker D, Leary RJ, Ptak J, Silliman N (2006). The consensus coding sequences of human breast and colorectal cancers. Science.

[5] Kaminker JS, Zhang Y, Watanabe C, Zhang Z (2007). CanPredict: a computational tool for predicting cancer-associated missense mutations. Nucleic Acids Res.

[6] Dees ND, Zhang Q, Kandoth C, Wendl MC, Schierding W, Koboldt DC, Mooney TB, Callaway MB, Dooling D, Mardis ER (2012). MuSiC: identifying mutational significance in cancer genomes. Genome Res.

[7] Tamborero D, Gonzalez-Perez A, Lopez-Bigas N (2013). OncodriveCLUST: exploiting the positional clustering of somatic mutations to identify cancer genes. Bioinformatics.

[8] Lawrence MS, Stojanov P, Polak P, Kryukov GV, Cibulskis K, Sivachenko A, Carter SL, Stewart C, Mermel CH, Roberts SA (2013). Mutational heterogeneity in cancer and the search for new cancer-associated genes. Nature.

[9] Torkamani A, Schork NJ (2009). Identification of rare cancer driver mutations by network reconstruction. Genome Res.

[10] Cheng F, Zhao J, Zhao Z (2016). Advances in computational approaches for prioritizing driver mutations and significantly mutated genes in cancer genomes. Brief Bioinform.

[11] Hamed M, Spaniol C, Zapp A, Helms V (2015). Integrative network-based approach identifies key genetic elements in breast invasive carcinoma. BMC Genomics.

[12] Hou JP, Ma J (2014). DawnRank: discovering personalized driver genes in cancer. Genome Med.

[13] Bashashati A, Haffari G, Ding J, Ha G, Lui K, Rosner J, Huntsman DG, Caldas C, Aparicio SA, Shah SP (2012). DriverNet: uncovering the impact of somatic driver mutations on transcriptional networks in cancer. Genome Biol.

[14] Cho A (2016). MUFFINN: cancer gene discovery via network analysis of somatic mutation data. Genome Biol.

[15] Kim YA (2015). MEMCover: integrated analysis of mutual exclusivity and functional network reveals dysregulated pathways across multiple cancer types. Bioinformatics.

[16] Ciriello G, Cerami E, Sander C, Schultz N (2012). Mutual exclusivity analysis identifies oncogenic network modules. Genome Res.

[17] Merid SK, Goranskaya D, Alexeyenko A (2014). Distinguishing between driver and passenger mutations in individual cancer genomes by network enrichment analysis. BMC Bioinform.

[18] Liu X, Liu ZP, Zhao XM, Chen L (2012). Identifying disease genes and module biomarkers by differential interactions. J Am Med Inform Assoc.

[19] Zhang W, Zeng T, Chen L (2014). EdgeMarker: identifying differentially correlated molecule pairs as edge-biomarkers. J Theor Biol.

[20] Vogelstein B, Papadopoulos N, Velculescu VE, Zhou S, Diaz LA, Kinzler KW (2013). Cancer genome landscapes. Science.

[21] Yano Y, Uematsu N, Yashiro T, Hara H, Ueno E, Miwa M, Tsujimoto G, Aiyoshi Y, Uchida K (2004). Gene expression profiling identifies platelet-derived growth factor as a diagnostic molecular marker for papillary thyroid carcinoma. Clinical Cancer Res.

[22] Siraj AK, Masoodi T, Bu R, Beg S, Al-Sobhi SS, Al-Dayel F, Al-Dawish M, Alkuraya FS, Al-Kuraya KS (2016). Genomic profiling of thyroid cancer reveals a role for thyroglobulin in metastasis. Am J Hum Genet.

[23] Varela I, Tarpey P, Raine K, Huang D, Ong CK, Stephens P, Davies H, Jones D, Lin ML, Teague J (2011). Exome sequencing identifies frequent mutation of the SWI/SNF complex gene PBRM1 in renal carcinoma. Nature.

[24] Leeman RJ, Lui VW, Grandis JR (2006). STAT3 as a therapeutic target in head and neck cancer. Expert Opin Biol Ther.

[25] Cancer Genome Atlas Research Network (2011). Integrated genomic analyses of ovarian carcinoma. Nature.

[26] Bebek G, Orloff M, Eng C (2011). Microenvironmental genomic alterations reveal signaling networks for head and neck squamous cell carcinoma. J Clin Bioinform.

[27] Lue HW, Cole B, Rao SA, Podolak J, Van Gaest A, King C, Eide CA, Wilmot B, Xue C, Spellman PT (2015). Src and STAT3 inhibitors synergize to promote tumor inhibition in renal cell carcinoma. Oncotarget.

[28] Giubellino A, Burke TR, Bottaro DP (2008). Grb2 signaling in cell motility and cancer. Expert Opin Ther Targets.

[29] Yip SS, Crew AJ, Gee JM, Hui R, Blamey RW, Robertson JF, Nicholson RI, Sutherland RL, Daly RJ (2000). Up-regulation of the protein tyrosine phosphatase SHP-1 in human breast cancer and correlation with GRB2 expression. Int J Cancer.

[30] Qiu W, Schönleben F, Li X, Su GH (2007). Disruption of transforming growth factor β-Smad signaling pathway in head and neck squamous cell carcinoma as evidenced by mutations of SMAD2 and SMAD4. Cancer Lett.

[31] Lin Y, Mallen-St Clair J, Wang G, Luo J, Palma-Diaz F, Lai C, Elashoff DA, Sharma S, Dubinett SM, St John M (2016). p38 MAPK mediates epithelial-mesenchymal transition by regulating p38IP and Snail in head and neck squamous cell carcinoma. Oral Oncol.

[32] Schiff BA, McMurphy AB, Jasser SA, Younes MN, Doan D, Yigitbasi OG, Kim S, Zhou G, Mandal M, Bekele BN (2004). Epidermal growth factor receptor (EGFR) is overexpressed in anaplastic thyroid cancer, and the EGFR inhibitor gefitinib inhibits the growth of anaplastic thyroid cancer. Clin Cancer Res.

[33] Howell GM, Hodak SP, Yip L (2013). RAS mutations in thyroid cancer. Oncologist.

[34] Fagin JA, Mitsiades N (2008). Molecular pathology of thyroid cancer: diagnostic and clinical implications. Best Pract Res Clin Endocrinol Metab.

[35] Gayther SA, Batley SJ, Linger L, Bannister A, Thorpe K, Chin SF, Daigo Y, Russell P, Wilson A, Sowter HM (2000). Mutations truncating the EP300 acetylase in human cancers. Nat Genetics.

[36] Pitt SC, Hernandez RA, Nehs MA, Gawande AA, Moore FD, Ruan DT, Cho NL (2016). Identification of novel oncogenic mutations in thyroid cancer. J Am Coll Surg.

[37] Hu ZQ, Ma R, Zhang CM, Li J, Li L, Hu ZT, Gao Q, Li WM (2015). Expression and clinical significance of tyrosine phosphatase SHP2 in thyroid carcinoma. Oncol Lett.

[38] Stransky N, Egloff AM, Tward AD, Kostic AD, Cibulskis K, Sivachenko A, Kryukov GV, Lawrence MS, Sougnez C, McKenna A (2011). The mutational landscape of head and neck squamous cell carcinoma. Science.

[39] Martin D, Abba MC, Molinolo AA, Vitale-Cross L, Wang Z, Zaida M, Delic NC, Samuels Y, Lyons GJ, Gutkind JS (2014). The head and neck cancer cell oncogenome: a platform for the development of precision molecular therapies. Oncotarget.

[40] Dent P (2013). FADD the bad in head and neck cancer. Cancer Biol Ther.

[41] Pattje W, Melchers L, Slagter-Menkema L, Mastik M, Schrijvers M, Gibcus J, Kluin P, Hoegen-Chouvalova O, Laan B, Roodenburg J (2013). FADD expression is associated with regional and distant metastasis in squamous cell carcinoma of the head and neck. Histopathology.

[42] Guo G, Gui Y, Gao S, Tang A, Hu X, Huang Y, Jia W, Li Z, He M, Sun L (2012). Frequent mutations of genes encoding ubiquitin-mediated proteolysis pathway components in clear cell renal cell carcinoma. Nat Genetics.

[43] Li H, Hes O, MacLennan GT, Eastwood DC, Iczkowski KA (2015). Immunohistochemical distinction of metastases of renal cell carcinoma to the adrenal from primary adrenal nodules, including oncocytic tumor. Virchows Archiv.

[44] Liu BH, Yu H, Tu K, Li C, Li YX, Li YY (2010). DCGL: an R package for identifying differentially coexpressed genes and links from gene expression microarray data. Bioinformatics.

[45] Benjamini Y, Hochberg Y (1995). Controlling the false discovery rate: a practical and powerful approach to multiple testing. J Roy Statist Soc Series B (Methodol).

[46] Diao H, Li X, Hu S, Liu Y (2012). Gene expression profiling combined with bioinformatics analysis identify biomarkers for Parkinson disease. PLoS One.

[47] Merid SK, Goranskaya D, Alexeyenko A (2014). Distinguishing between driver and passenger mutations in individual cancer genomes by network enrichment analysis. BMC Bioinform.

[48] Kanehisa M, Goto S, Kawashima S, Nakaya A (2002). The KEGG databases at GenomeNet. Nucleic Acids Res.

[49] Ashburner M, Ball CA, Blake JA, Botstein D, Butler H, Cherry JM, Davis AP, Dolinski K, Dwight SS, Eppig JT (2000). Gene Ontology: tool for the unification of biology. Nat Genet.

[50] Hanahan D, Weinberg RA (2011). Hallmarks of cancer: the next generation. Cell.

[51] Huang DW, Sherman BT, Tan Q, Collins JR, Alvord WG, Roayaei J, Stephens R, Baseler MW, Lane HC, Lempicki RA (2007). The DAVID gene functional classification tool: a novel biological module-centric algorithm to functionally analyze large gene lists. Genome Biol.

[52] Bader GD, Hogue CW (2003). An automated method for finding molecular complexes in large protein interaction networks. BMC Bioinform.

