# Supplementary material for: Integrating omics data and protein interaction networks to prioritize driver genes in cancer
Source: Oncotarget. 2017 Jul 22;8(35):58050–60. doi: 10.18632/oncotarget.19481 (PMC5601632; doi:10.18632/oncotarget.19481)
Supplement: Supplementary file 1 [file oncotarget-08-58050-s001.pdf]

Integrating omics data and protein interaction networks to prioritize driver genes in cancer

SUPPLEMENTARY MATERIALS

Supplementary Table 1: Ranked list of candidate driver genes for HNSC, KIRC, and THCA dataset, respectively. Rank: rank of the gene according to our method, gene: gene symbol, driver gene score: the score of driver gene calculated by our method.

See Supplementary File 1

Supplementary Table 2: KEGG pathway enrichment analysis for all driver genes identified from THCA, KIRC and HNSC respectively.

See Supplementary File 2
